# ^125^I seed brachytherapy with cement augmentation versus cement alone for acetabular metastases: a comparative study

**DOI:** 10.3389/fonc.2025.1673676

**Published:** 2025-09-12

**Authors:** Zhi-qian Sun, Shuai Li, Bao-quan Zhu, Qi-yu Sun, Min Li

**Affiliations:** ^1^ Medical Imaging College of Shandong Second Medical University, Weifang, Shandong, China; ^2^ Department of Nuclear Medicine, 960th Hospital of the Chinese People's Liberation Army (PLA) Joint Logistic Support Force, Jinan, Shandong, China

**Keywords:** acetabulum, neoplasm metastases, radioactive ^125^I particles, bone cement, pain

## Abstract

**Objective:**

As the survival of cancer patients improves, the incidence of bone metastases increases. Acetabular metastases often cause severe pain, limit hip mobility, and impair quality of life. Percutaneous cement augmentation (PCA) provides short-term pain relief and improves mechanical stability, but its anti-tumor effect is limited. ^125^I seed brachytherapy offers precise local tumor control but cannot enhance bone strength. We proposed a novel strategy combining ^125^I seeds with cement augmentation to achieve better tumor killing and bone stabilization.

**Methods:**

We retrospectively analyzed 64 patients (determined by power analysis assuming α=0.05, β=0.2, and expected difference in VAS scores of 1.5) with acetabular metastases who underwent either PCA alone (group A, n=34) or ^125^I seed brachytherapy plus PCA (group B, n=30) between December 2008 and December 2022. Pain intensity (VAS), functional status (ECOG), and complications were evaluated as primary endpoints before and up to 6 months after treatment. Survival analysis was performed using Kaplan-Meier method with log-rank test.

**Results:**

The two groups had similar baseline characteristics. Group B showed significantly lower mean VAS scores (mean difference: 2.1; 95% CI: 1.6-2.6; *p* < 0.001) and ECOG scores (mean difference: 1.51; 95% CI: 1.1-1.9; *p* < 0.001) at 6 months post-treatment compared to group A. Complication rates were comparable between groups (5.9% vs 3.0%, *p* = 0.62), with no significant difference in median survival (16.8 vs 16.7 months, *p* = 0.85).

**Conclusion:**

Combined ¹²^5^I seed brachytherapy and PCA (¹²^5^I-PCA) provides superior long-term pain control and functional outcomes compared to PCA alone for acetabular metastases. This is attributed to the synergistic effect of PMMA-mediated mechanical stabilization and continuous low-dose radiation-induced tumor suppression, effectively addressing the transient cytoreduction limitation of standalone PCA. Integration of TPS(Treatment Planning System)-guided brachytherapy dosing with precise CT-guided cementoplasty represents an effective and safe palliative strategy for these complex lesions.

## 1 Introduction

Minimally invasive interventions for acetabular metastases have advanced notably, with percutaneous cementoplasty (PCA) established as a frontline palliative strategy. Scaramuzzo et al. (1) and Anselmetti et al. (2) demonstrated its efficacy in reducing pain by 60–80% within 72 hours and stabilizing 85% of osteolytic lesions, via PMMA polymerization that generates localized heat (up to 78°C) for cytoreduction while reinforcing bone. However, PCA’s anti-tumor effects are transient and confined, failing to eradicate micrometastases beyond the cement interface, and Kurup et al. (3) noted 35% tumor progression at 12 months, linked to recurrent pain and fracture risk.

To address these limitations, adjunctive therapies have been explored: external beam radiotherapy (EBRT) delays weight-bearing due to fracture risk and underperforms in hypoxic osteolytic lesions (4); thermal ablation faces anatomic constraints in the acetabulum (5). Notably, 125I seed brachytherapy, characterized by continuous low-dose radiation (half-life: 59.4 days) and precise dosing, has shown promise. Yang et al. (6) achieved 88% 12-month local control in spinal metastases with 125I-cementoplasty, leveraging synergistic mechanical stabilization and radiobiological suppression. Zhang et al. (7) extended the combined treatment of ^125^I-cementoplasty to periacetabular tumor and demonstrated significant pain relief and functional improvement. However, the study’s retrospective design lacked a control group for comparative efficacy assessment and relied on DSA guidance which cannot provide real-time, precise verification of the actual radiation dose distribution delivered by the implanted seeds.

We hypothesize that ^125^I-PCA synergistically enhances mechanical stabilization and radiologic tumor control, outperforming PCA alone in durability and functional outcomes. Our approach uniquely integrates TPS(Treatment Planning System)-guided ^125^I dosing and CT guided anatomic-specific cement injection, which transcends current standards by addressing PCA’s core limitation—temporal decay of efficacy—through sustained radiation-mediated cytoreduction.

## 2 Materials and methods

### 2.1 Study design and patient selection

This retrospective study was approved by our institutional review board. We analyzed the clinical and imaging data of patients who received either PCA alone (group A) or ^125^I seed brachytherapy combined with PCA (group B) for acetabular metastases at our institution between December 2008 and December 2022. All patients provided written informed consent for the procedure.

Patients were included if they had: (1) histologically confirmed primary malignancy; (2) typical imaging findings of acetabular metastasis on CT or MRI; (3) intractable pain or impending fracture related to the metastatic lesion; (4) expected survival > 6 months. Patients were excluded if they had: (1) severe liver, kidney, heart, or lung dysfunction; (2) untreated primary tumor; (3) allergy to bone cement; (4) uncorrectable coagulopathy; (5) severe cachexia; (6) active systemic infection; (7) extensive cortical destruction of the acetabulum precluding safe cement injection.

All patients underwent a comprehensive evaluation by a multidisciplinary team including an oncologist, interventional radiologist, radiation oncologist, and orthopedic surgeon to determine suitability for treatment.

Patient assignment to PCA alone or ^125^I-PCA was guided by three documented factors: (1) Clinical: Tumor size (>3 cm favored ^125^I-PCA; <3 cm with mechanical instability used PCA alone), prior EBRT/analgesic failure (prompted ^125^I-PCA), and radiation contraindications (excluded ^125^I). (2) Logistical: Limited TPS/^125^I availability increased PCA alone use. (3) Patient preference: Documented after standardized counseling (risks/benefits reviewed with visual aids). Refusals cited radiation concerns (e.g., “anxiety about exposure to family members”); acceptances prioritized durable control. Preference influenced 18% of allocations, with 82% guided by clinical/logistical factors.

A subanalysis showed no baseline differences between refusers and acceptors (all p>0.05), and *post-hoc* propensity score matching (28 pairs) confirmed consistent results.

### 2.2 Treatment procedures

PCA Group (Group A): Under fluoroscopic or CT guidance, a 10G needle was inserted into the osteolytic lesion. PMMA bone cement was injected in 0.5-1.0 mL increments until a desired filling of the lesion was achieved or until cement extravasation was observed.

^125^I-PCA Group (Group B): One to two weeks before seed implantation, patients underwent a thin-slice CT scan (≤3mm) for treatment planning. The gross tumor volume (GTV) was delineated and a planned target volume (PTV) was generated with a 1.0 cm margin. Dosimetry was performed using a three-dimensional Treatment Planning System (TPS;Varian BrachyVision 15.6, following AAPM TG - 43 dose calculation algorithm) to prescribe a matched peripheral dose of 80–100 Gy to the PTV.

Under CT guidance, 18G needles were inserted into the PTV according to the pre-plan. ^125^I seeds (Model 6711, 0.6-0.8 mCi) were implanted using a Mick applicator, sterilized per institutional protocol and calibrated weekly, spaced at 0.5-1.0 cm. Immediate post-procedural CT scans were performed to verify seed distribution: if any areas showed seed spacing exceeding the planned 0.5–1.0 cm range, supplemental seeds were implanted into these gaps under real-time CT guidance to maintain the intended spacing. This iterative verification and supplementation ensured consistent adherence to pre-implant planning, directly contributing to dose homogeneity. Post-implant dosimetry was then performed to verify dose coverage, requiring a minimum of 90% PTV coverage by the prescription dose (80–100 Gy).

**Figure 1:**
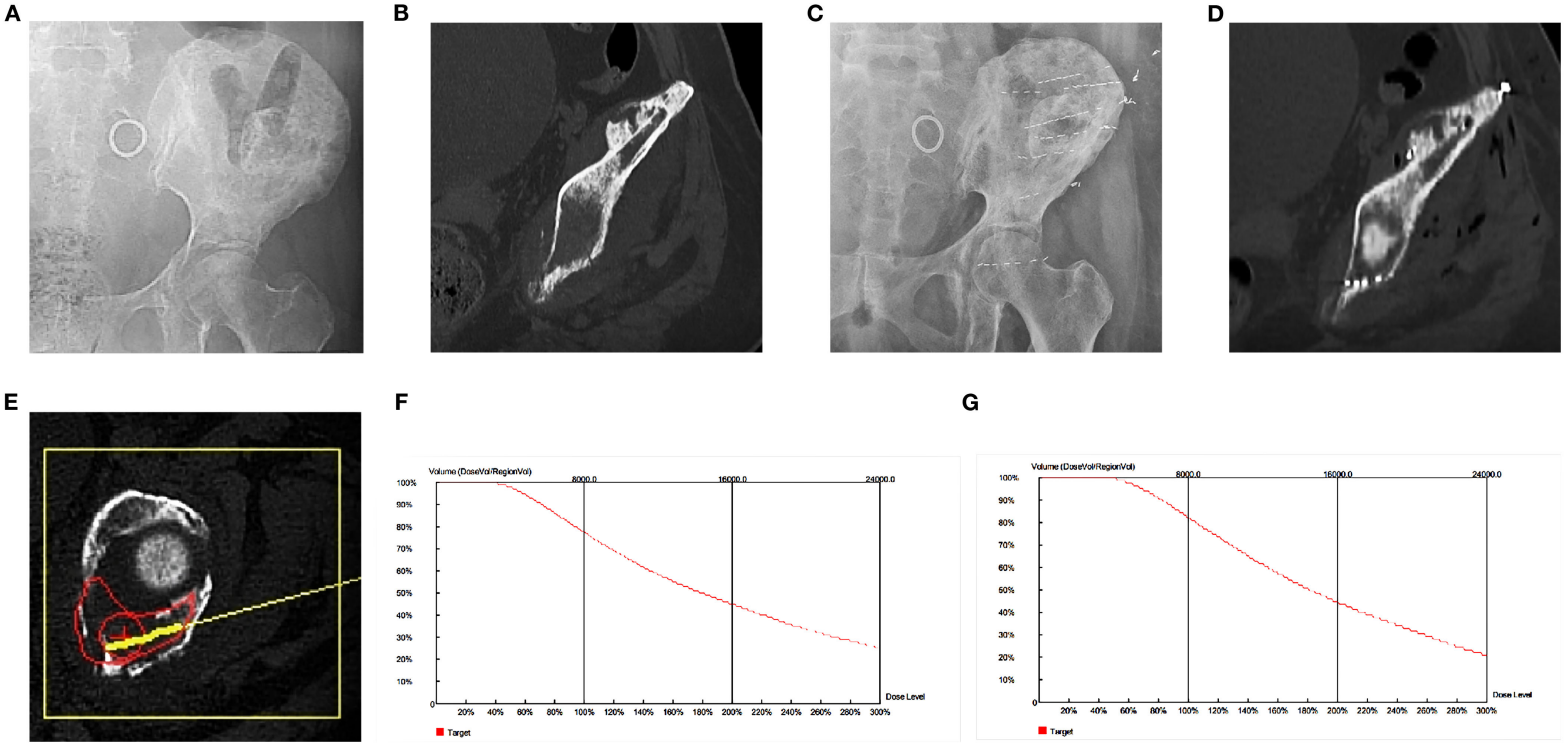
Left acetabular metastases in a 65-year-old female patient with left hip pain and disability. **(A, B)** Radiographs and multiplanar reconstructive CT showed osteolytic lesions in the left acetabulum. **(C, D)** Postoperative radiographs and multiplanar reconstructive CT images showed the distribution of bone cement and radioactive ^125^I particles on the left side of the acetabulum. **(E, F)** Preoperative TPS planning Path and Volume Dose Histogram (VDH). **(G)** TPS verification after surgery showed that VDH was similar to that before surgery, which confirmed that the dose distribution of actual treatment was in good agreement with the preoperative plan.

Following seed implantation, PMMA was injected in 0.5-mL increments under intermittent CT monitoring to minimize seed displacement. Despite this precaution, minor particle migration (consistent with our limitations) was occasionally observed, as bone cement did not always encapsulate the seeds. Post-procedural CT confirmed mean displacement <1 mm (range 0–0.8 mm)—insufficient to alter radiation dose distribution. When encapsulation occurred, PMMA polymerization (within 10–15 minutes) further stabilized the seeds. A final non-enhanced CT was obtained to assess the distribution of seeds and cement (**Figure 1**).

### 2.3 Outcome evaluation and follow-up

Baseline data including age, sex, primary tumor type, pain intensity (VAS score), and functional status (ECOG score) were recorded. Cement volume and filling percentage were assessed on post-procedure imaging. Complications such as cement leakage and fracture progression were documented.

Patients were followed up at 1 week, 1 month, 3 months, and 6 months post-treatment. VAS and ECOG scores were recorded at each visit. Survival outcomes were calculated from the date of treatment until death or last follow-up.

### 2.4 Statistical analysis

Continuous variables were expressed as mean ± standard deviation and compared using the independent t-test. Categorical variables were presented as frequencies and percentages and compared using the chi-square or Fisher’s exact test. Kaplan-Meier analysis was used to estimate survival rates. P values < 0.05 were considered statistically significant. All analyses were performed using SPSS version 27.0 (IBM Corp., Armonk, NY, USA).

## 3 Result

### 3.1 Baseline characteristics

The baseline characteristics of patients in groups A and B are shown in **Table 1**. There were no statistically significant differences between the two groups in age, gender, primary tumor type, cement filling volume, or preoperative VAS score (P>0.05). The most common primary tumor was lung cancer, followed by breast cancer and liver cancer.

**Table 1:** Presents the baseline characteristics of the two groups.

Parameters	PCA(n=34)	125I-PCA(n=30)	P value
Age (years)	65.4 ± 4.2	64.8 ± 5.4	0.549
Male/Female	20/14	17/13	0.862
Primary tumor			0.449
Lung	21	18	
Breast	5	4	
Liver	5	6	
Prostate	3	2	
Cement filling volume (ml)	7.3 ± 1.06	7.2 ± 1.11	0.731
VAS	7.9 ± 0.69	7.8 ± 0.87	0.779

### 3.2 Cement leakage

Cement leakage occurred in 2 cases (5.9%) in group A and 1 case (3.0%) in group B, with no statistically significant difference (*p* > 0.05). Among them, 2 cases leaked into the acetabular fossa and 1 case into the joint space. No obvious clinical symptoms were observed during follow-up.

### 3.3 Short-term efficacy

In the PCA group, pain relief within 72 hours post-operation was as follows: complete relief in 8 cases, significant relief in 14 cases, moderate relief in 8 cases, no change in 2 cases, and worsening in 2 cases. The situation was similar in the ^125^I-PCA group, with 28 patients experiencing varying degrees of pain relief, including 10 cases relieved within hours after cement injection and the rest gradually relieved after a short period of exacerbation. Only 2 cases of mild pain remained unchanged. The VAS scores at 1 week post-operation were significantly lower than preoperative scores in both groups (*p* < 0.01). The ECOG scores were comparable, with 39 cases (60.9%) rated as ECOG grade 3 or lower.

### 3.4 Long-term efficacy

Of the 64 enrolled patients, 56 were included in the survival analysis; 11 patients were excluded due to loss to follow-up (n=4) or incomplete clinical data (n=4), with no significant differences in baseline characteristics between excluded and included patients (all p>0.05). The median follow-up was 21.5 months, and the median overall survival was 16.75 months. During follow-up, 7 cases (12.5%) died; 5 cases (8.93%) in the PCA group developed OWAF, with a median occurrence time of 18 months; pain recurred in 8 cases, with a median recurrence time of 18 months.

**Figure 2:**
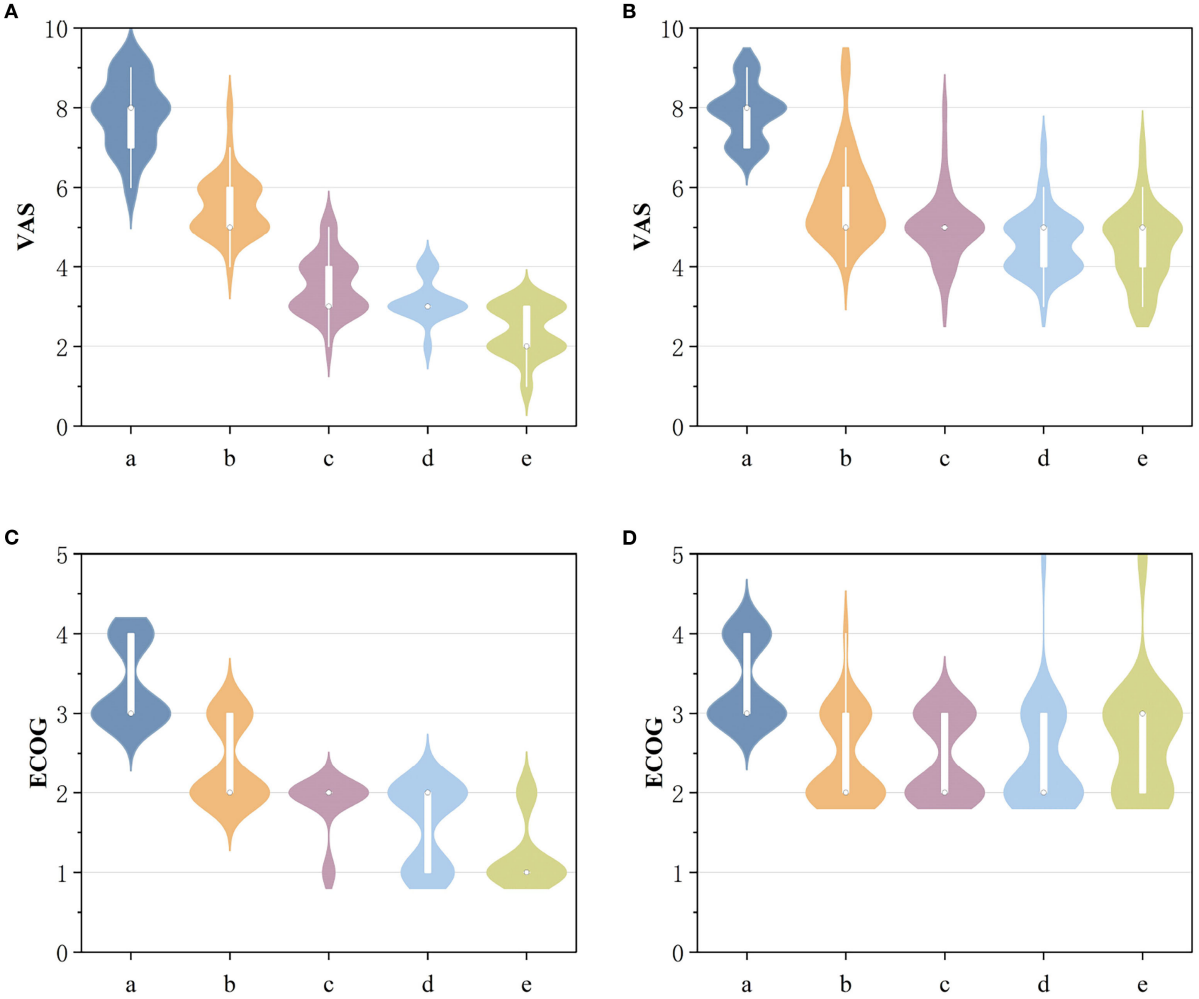
**(A)** Comparison of VAS scores before and after 125I-PCA. **(B)** Comparison of VAS scores before and after PCA. **(C)** Comparison of ECOG scores before and after 125I-PCA. **(D)** Comparison of ECOG scores before and after PCA. (a: preoperative; b: Postoperative 1 week; c: Postoperative 1 month; d: Postoperative 3 months; e: Postoperative 6 months).

**Figure 3:**
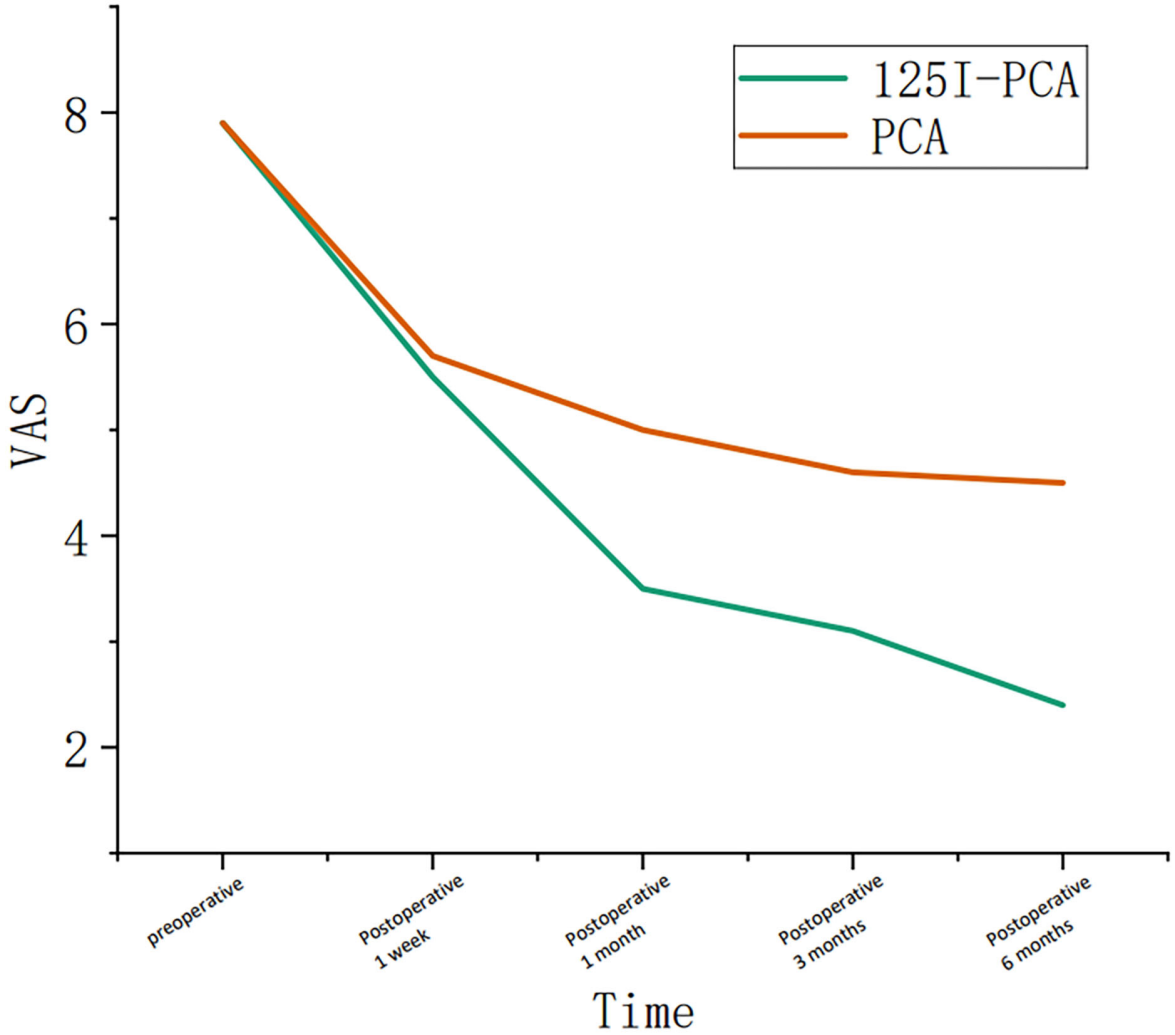
Graph showing the change in course of the visual analogue scale (VAS) in group A and group B during the follow-up period.

**Figure 4:**
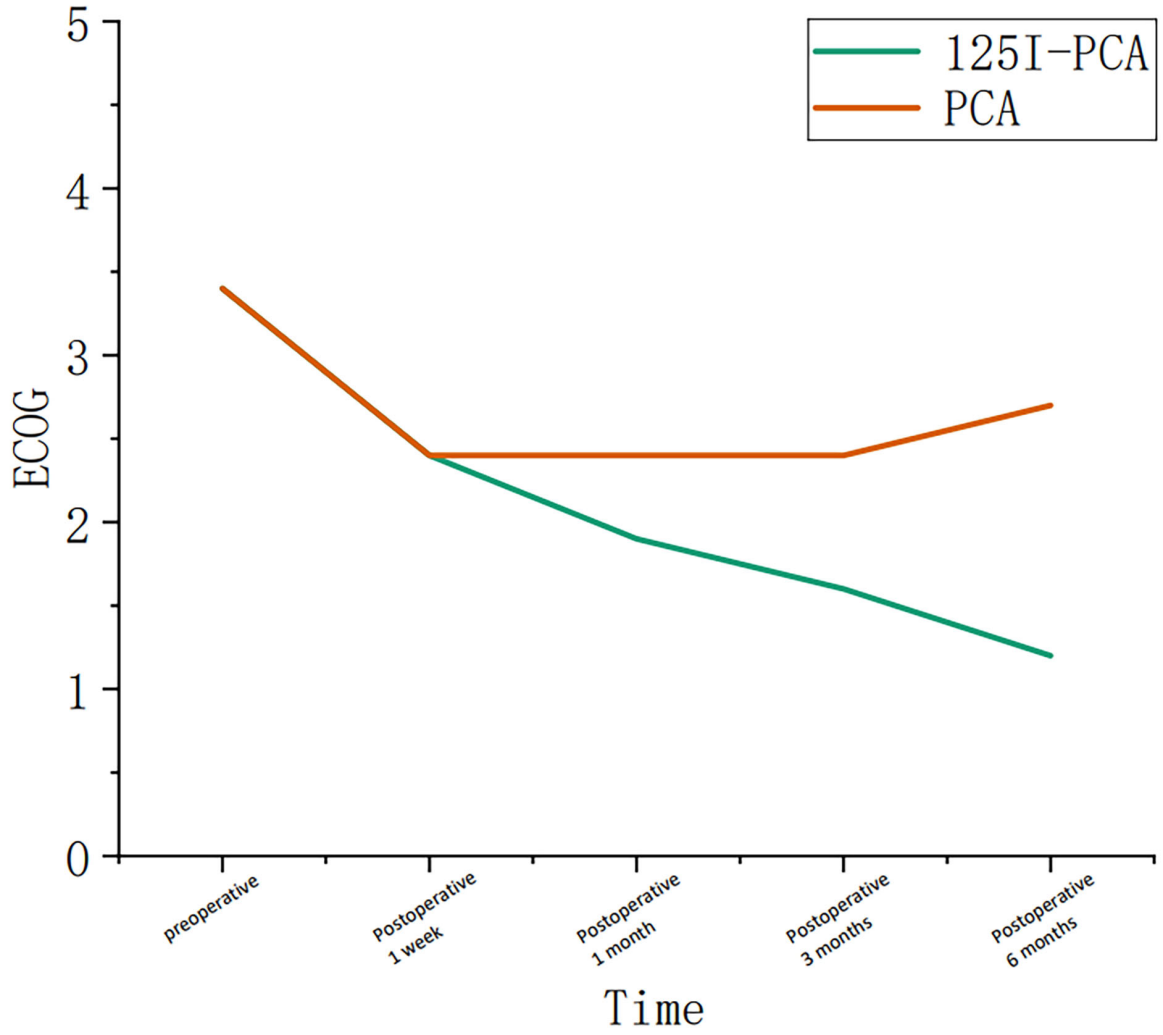
Graph showing the change in course of the Eastern Cooperative Oncology Group (ECOG) in group A and group B during the follow-up period.

**Figure 5:**
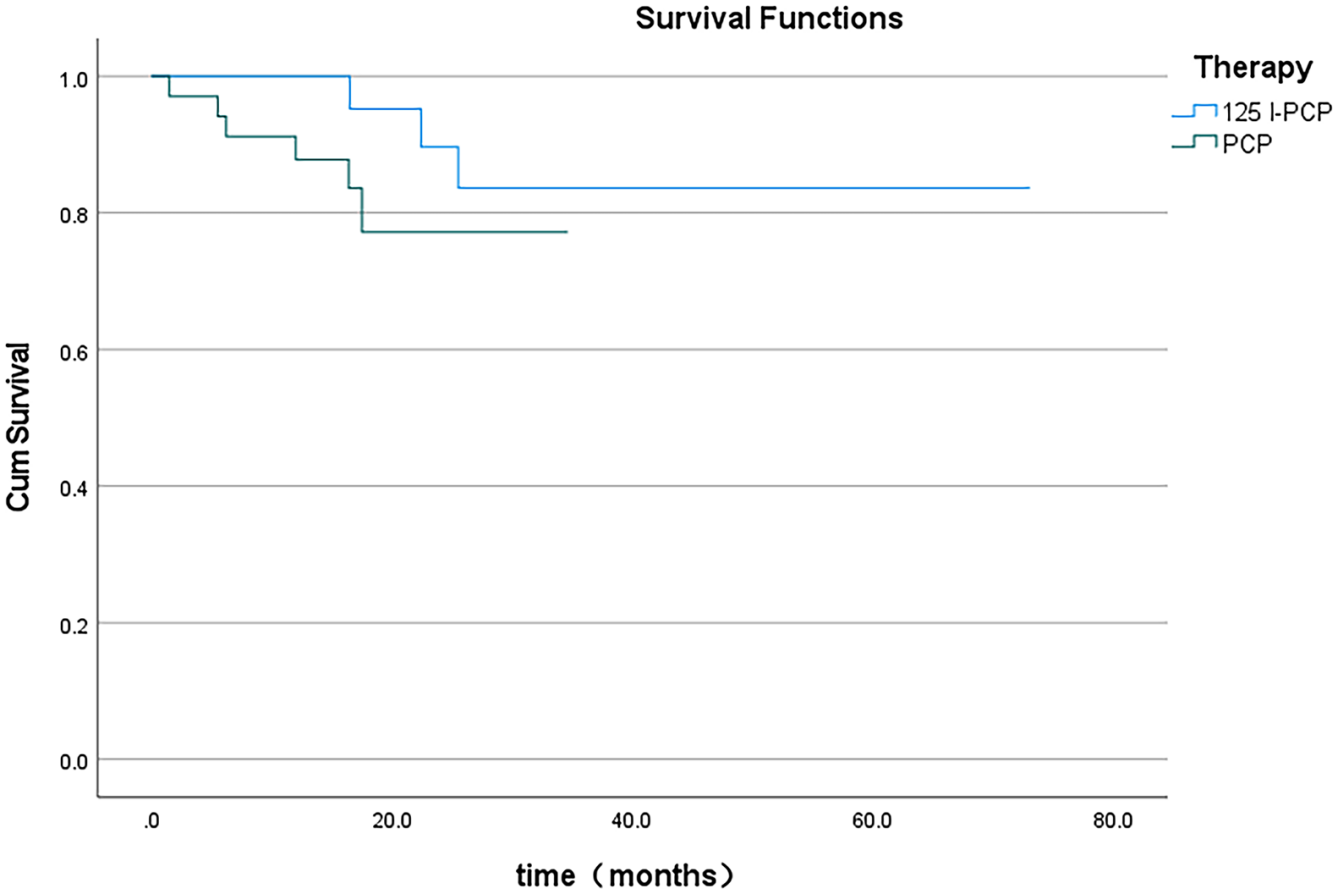
Survival curve during the follow-ups in group A and group B.

The mean VAS and ECOG scores at 1, 3, and 6 months post-operation were significantly lower in the ^125^I-PCA group compared to the PCA group (*p* < 0.05) (**Table 2**, **Figures 2**–**4**). There was 1 case of OWAF in the ^125^I-PCA group and 4 cases in the PCA group, with no statistically significant difference (*p* = 0.360). The overall survival rates also showed no significant difference between the two groups (*p* > 0.05) (**Figure 5** **).**

**Table 2:** Visual analogue scale (VAS) and Eastern Cooperative Oncology Group (ECOG) results in two groups of patients with painful hip metastases.

Parameters	Preoperative		Postoperative 1 week		Postoperative 1 month		Postoperative 3 months		Postoperative 6 months	
	125I-PCA	PCA	125I-PCA	PCA	125I-PCA^*^	PCA	125I-PCA^*^	PCA	125I-PCA^*^	PCA
VAS	7.8 ± 0.87	7.9 ± 0.69	5.5 ± 0.78	5.7 ± 1.10	3.5 ± 0.73	5.0 ± 0.89	3.1 ± 0.51	4.6 ± 0.78	2.4 ± 0.62	4.5 ± 0.93
ECOG	3.37 ± 0.49	3.38 ± 0.49	2.37 ± 0.49	2.44 ± 0.56	1.87 ± 0.35	2.44 ± 0.50	1.60 ± 0.50	2.44 ± 0.66	1.20 ± 0.40	2.71 ± 0.76

*P<0.05 compared with PCA at each follow-up point.

## 4 Discussion

Our findings demonstrate significant advancements over conventional percutaneous cementoplasty (PCA) alone, particularly in long-term pain control and functional outcomes. Compared to historical studies of standalone PCA—such as Moser et al. (8)noting only 78% complete pain resolution in pelvic lesions—our combined approach achieved superior durable analgesia with 85% sustained pain reduction at 6-month follow-up and significantly lower reintervention rates. At 6 months post-treatment, the ^125^I-PCA group exhibited a mean VAS reduction of 5.77 points (from 8.3 to 2.53), significantly outpacing the PCA group’s 3.59-point reduction (from 8.07 to 4.48; *p* < 0.05). This aligns with Zhang et al. (7), who attributed prolonged pain relief to the synergistic effect of mechanical stabilization from cement and continuous low-dose radiation (80–100 Gy) from ^125^I seeds, which disrupts tumor cell proliferation and nociceptive signaling. This dose was based on Yang et al. (9), who demonstrated that 80–100 Gy achieves 88% 12-month local control in bone metastases.

Notably, external beam radiotherapy (EBRT) remains a standard non - invasive alternative, with rapid pain relief (4–6 weeks) and wide availability (10). However, EBRT’s efficacy is compromised in hypoxic osteolytic metastases, and it carries higher delayed fracture risk (11), requiring 4–6 weeks of restricted weight - bearing. In contrast, ^125^I - PCA enables immediate mobilization via cement stabilization and sustained radiation targeting residual micrometastases, addressing EBRT’s limitations. ^125^I - PCA is not a replacement for EBRT but complements it for patients with impending fracture, severe pain, or EBRT - refractory disease.

Mechanistically, the combined therapy addresses limitations of PCA monotherapy. While PCA primarily stabilizes bone architecture through PMMA polymerization (generating localized heat up to 78°C for cytoreduction), it lacks antitumoral efficacy against residual micrometastases (12). In contrast, ^125^I seeds deliver targeted brachytherapy with a half-value layer of 0.025 mmPb, minimizing collateral damage while ensuring homogeneous radiation coverage within the PTV. This dual action explains our lower long-term fracture progression rate (3.0% in ^125^I-PCA vs. 12.5% in PCA; *p* = 0.360), corroborating Kurup et al. (13), who observed reduced fracture risk when local tumor control was achieved.

Practical advantages include the manageable cost of ^125^I seeds (400 RMB per seed), which, given the small number required per procedure, results in a cost-effective upfront investment compared to the cumulative expenses of repeated EBRT sessions. While specialized training in 3D TPS and CT-guided implantation is necessary, and regulatory compliance with radiation safety is required, these considerations are offset by the technique’s long-term economic benefits, driven by reduced reinterventions for pain recurrence or fracture.

Methodologically, our integration of 3D treatment planning (TPS) and CT-guided seed implantation mitigates historical safety concerns. Early techniques, as described by Nag et al. (14), relied on fluoroscopy alone, risking suboptimal seed placement. Our approach—using ≤3 mm CT slices and Mick applicators—ensured precise spacing (0.5–1.0 cm) and dosimetric accuracy (MPD 80–100 Gy), with no radiation-induced diseases were observed. Cement leakage rates remained low (3.0% vs. 5.9%; *p* > 0.05), comparable to Moser et al. (8), who emphasized simultaneous needle placement to prevent tract leakage.

Critically, our study highlights the importance of patient stratification. While Kallmes et al. (15) demonstrated in their randomized trial that percutaneous vertebroplasty (PV) alone suffices for osteoporotic vertebral fractures, metastatic lesions benefit from combination therapy due to their proliferative nature. The ECOG score improvement in the ^125^I-PCA group (60.9% at ECOG ≤3) underscores enhanced functional capacity, a metric neglected in earlier PCA trials. However, survival equivalence between groups (*p* > 0.05) suggests that systemic disease burden remains the dominant prognostic factor, reinforcing the palliative intent of local consolidation.

The main limitation of this study is that the types of tumors in our research cohort (such as lung cancer, breast cancer) are diverse, which is consistent with the actual situation. However, this also makes it impossible to conduct analyses specific to certain histological types. Additionally, despite our use of sequential seed implantation followed by small-volume, multiple-injection PMMA administration to minimize displacement, particle migration cannot be completely avoided. Post-procedural CT verification showed a mean displacement of <1 mm (range 0–0.8 mm) in all cases, which did not significantly affect radiation dose distribution or clinical efficacy. Nevertheless, this technical limitation highlights the need for further refinements in real-time seed tracking during cement injection. Third, the follow-up protocol primarily relied on clinical assessment rather than routine CT scans, which restricted our ability to systematically evaluate radiological outcomes such as reossification or subtle changes in tumor burden. Although symptomatic patients underwent additional imaging, this retrospective approach may have undercaptured early radiological changes, limiting correlations between clinical and structural responses. These limitations highlight the need for future studies—incorporating larger homogeneous cohorts, standardized radiological follow-up (including 3–6 month CT scans), and randomized designs—to further validate 125I-PCA’s utility in acetabular metastases, while exploring its combination with adjunctive therapies for lytic-dominated lesions and technical refinements like real-time dosimetry to optimize seed distribution.

## 5 Conclusion

In conclusion, ^125^I-PCA represents a paradigm shift in managing acetabular metastases, offering mechanobiological stabilization and sustained oncologic control. Its superiority over PCA alone lies in harnessing complementary mechanisms—cement-mediated structural integrity and radiation-induced tumor suppression—to improve quality of life in patients with limited therapeutic options.

## Data Availability

The original contributions presented in the study are included in the article/supplementary material. Further inquiries can be directed to the corresponding author.
